# Diagnostic In Vivo Sensing of COVID-19 Antibody Detection Using DNA-Linking Graphene Oxide Synthetic Mimic Skin Tattoo Probes

**DOI:** 10.3390/microorganisms13020354

**Published:** 2025-02-06

**Authors:** Kyung Lee, Dong Ho Kim, Sihyun Jun, Yeseul Oh, Ye Jun Oh, Seo Jun Lee, Keumsook Kim, Suw Young Ly

**Affiliations:** 1 Biosensor Research Institute, Seoul National University of Science & Technology, Seoul 01811, Republic of Koreasuwyoung@snut.ac.kr (S.Y.L.); 2 University of Michigan College of Pharmacy, 428 Church St, Ann Arbor, MI 48109, USA; 3 Department of Radiological & Clinical Research, Korea Cancer Center Hospital, Korea Institute of Radiological and Medical Sciences, Seoul 01812, Republic of Korea

**Keywords:** graphene, DNA immobilized, influenza, COVID-19, virus, electrochemical, in vivo assay, tattoo sensor

## Abstract

COVID-19 antibody detection is dependent on highly specialized, time-consuming techniques, such as PCR separation, DNA amplification, and other methods such as spectrophotometric absorption. For these reasons, specialized technical training is necessary because individual diagnostic treatment is difficult. We have attempted to perform rapid sensing with a detection time of only 30 s. Additionally, we used a wearable multi-layer graphene oxide nanocolloid synthetic skin tattoo probe assay for influenza and COVID-19 virus detection with an electrochemical antigen–antibody redox ionic titration circuit. Cyclic voltametric−2 V~2.0 V potential windows were used. The diagnostic detection limit was determined using stripping anodic and cathodic amplifiers, and the working probe was fabricated with a graphene molecule structure with a virus antigen-immobilized amplifier. With redox potential strength obtained within −1.0 V~−1.3 V ionic activity, anodic and cathodic current linearly increased in the phosphate-buffered saline 5 mL electrolyte. The results indicate that instant detection was enabled via individual and wearable tattoo sensors.

## 1. Introduction

The COVID-19 pandemic was first identified in December 2019 [1]. After the death toll exploded [2], the WHO declared an international public health emergency in January 2020. After more deaths occurred [3], the status was upgraded to a pandemic in March 2020. The number of confirmed cases exceeded 1.884 billion, and the death toll exceeded 4.06 million.

Symptoms of COVID-19 vary and may include the following: fever, cough, sore throat, nasal congestion or runny nose, shortness of breath or difficulty breathing, chills or recurrent shaking with chills, new loss of smell or taste, fatigue, muscle pain, headache, nausea or vomiting, and diarrhea. If symptoms do appear, they usually appear about 2 to 10 days (incubation period) after infection. In the case of omicron mutations, symptoms usually appear within 2 to 4 days. Many infected people have no symptoms or only mild illness. People with COVID-19 are at increased risk of severe illness and death. For most people, symptoms resolve within about a week. However, in some people, symptoms last longer, sometimes accompanied by shortness of breath, coughing, and extreme fatigue, and remain active for several weeks. Long-lasting illness is more common in people who originally had a severe illness, although even people with mild illness can experience persistent symptoms. For 25–50% of people infected with COVID-19, symptoms persist for several months. This phenomenon has been referred to by various names, including long-term acute COVID-19 post-infection syndrome or condition. Sometimes, the elderly and others with this disease even die. The existing diagnostic technologies are time-consuming to implement.

In addition to respiratory disease, which can be severe and cause death, other serious complications include the following:

- Heart disease, including arrhythmia, myocardial disease, and acute heart injury.
- Coagulation disorders, including blood clots and bleeding in the small and large blood vessels.
- Guillain–Barré syndrome (rare).

A rare complication called multisystem inflammatory syndrome in children (MIS-C), which may be related to COVID-19, has been reported. Symptoms of this syndrome are similar to the rare disease Kawasaki disease and may include fever, abdominal pain, and rash. Vaccination can prevent the development of MIS-C. Similar complications (multisystem inflammatory syndrome in adults, MIS-A) have been reported in young and middle-aged adults. Here, COVID-19 virus detection techniques are related to PCR molecule amplification techniques, such as neutralizing antibody titers [4], a Photon Excitation Assay [5], and the polymerase chain reaction method [6]. All diagnostic methods are time-consuming, and some individual cases cannot be detected. For this reason, we have developed a technology that can be easily analyzed by anyone, anytime, using rapid and simple analytical sensing methods. Other examples of similar sensing methods include the picomolar assay [7] of in vivo gastric cancer cells [8], trace-metal detection in distilled alcoholic drinks or food [9], and the detection of trace cobalt ions in in vivo plant cells [10]. Such methods require skills involving simple working electrode modifications for the implanted neurotransmitter stimulation assay [11] and diagnostics of patient fluid sensing [12], which can be assayed using ex vivo fluid [13]. Furthermore, voltammetric ionic detections are usable for human hepatitis B virus detection in non-treated blood [14] by using DNA-linked carbon nanotube biosensing of helicobacter pylori bacterial infections [15]. Using the more sophisticated circuit of spectrometric [16] infrared photodiode [17] tattoo human skin [18] sensing is possible for the real-time analysis of neurotransmitters in the brain [19] and neurovascular implementation of the signaling pathways of a biocircuit [20]. These previously developed rapid modifications can be applied to COVID-19 antibody detection using self-diagnostic technology.

## 2. Materials and Methods

In this section, we will describe the diagnostic assays of the WHO standard protocols.

The conventional WHO protocols of PCR DNA detection, agarose gel electrophoresis separation, and spectrometric absorption methods are time-consuming and require sophisticated skills. So, highly trained master’s and doctoral technicians are needed for these methods. The necessary equipment, sequence, and reagents indicated are here.

Protocol 1

Conventional RT-PCR analyses for the matrix gene of influenza type A viruses.

Agarose gel electrophoresis of RT-PCR products. Prepare agarose gel, load PCR products and molecular weight markers, and run according to standard protocols. Visualize the presence of markers under UV light. An example of the material required and the procedure is given below.

Materials required:

- Agarose gel casting tray and electrophoresis chamber;
- Power supply and electrode leads;
- UV light box (λ = 302 nm);
- Camera and Polaroid^®^ film or any digital gel documentation system;
- Adjustable pipettes;
- 2% agarose gel in 1× TAE buffer;
- 1× TAE buffer
- Ethidium bromide (10 mg/mL);
- 6× Gel loading buffer (GLB);
- Molecular weight marker.

Also, the next steps are expensive and required skilled personnel.

Procedure A

Casting the agarose gel

1. Place a gel casting tray onto a gel casting base. Insert a comb and level the base.
2. Prepare 2% agarose by weighing out 4 g of agarose powder and dissolving it in 200 mL 1× TAE buffer.
3. Dissolve the agar by heating it in a microwave oven.
4. Cool the melted agarose to about 60 °C, then add 10 µL of ethidium bromide.
5. Pour the melted agarose into the gel casting tray.
6. Allow the gel to solidify at room temperature.
7. Remove the comb from the frame.
8. Place the tray into the electrophoresis chamber with the wells at the cathode side.
9. Fill the buffer chamber with 1× TAE at a level that can cover the top of the gel.

Also, sample treatment is complicated and requires skilled personnel.

B. Sample loading

1. Add 5 µL of the gel loading buffer to each PCR tube.
2. Load the molecular weight marker to the first well of the agarose gel.
3. Pipette 15 µL of the PCR product in the gel loading buffer to the gel.
4. Close the lid on the chamber and attach the electrodes. Run the gel at 100 V for 30–35 min.
5. Visualize the presence of the marker and PCR product bands with UV light.
6. Document the gel picture by photographing it. Interpret the results [21].

Interpretation of results

The size of the PCR products obtained should be compared with the expected product size. Tests should always be run with a positive control https://www.who.int/initiatives/global-influenza-surveillance-and-response-system (accessed on 1 January 2024).

Table 1 shows the CDC 2019 novel coronavirus (nCoV) approved rRT-PCR detection primer name of the DNA sequence. This contains the primers for the *N1*, *N2*, *N3*, and *RP* genes. A specific assay of the amplified fragments is employed using fluorescent spectro-absorption labeled probe oligonucleotides that are complementary to the target sequence numbers. The qualitative tests are described at https://www.fda.gov/medical-devices/emergency-situations-medical-devices/emergency-use-authorizations (accessed on 30 February 2023).

A sequencing protein was used to antigen immobilize the DNA working probe and to diagnose the standard fabrications. So, we have simplified the skin sensor which was prepared as follows.

## 3. Mimic Skin Synthesis

### 3.1. Molecular Structure of Standards

**Figure 1:**
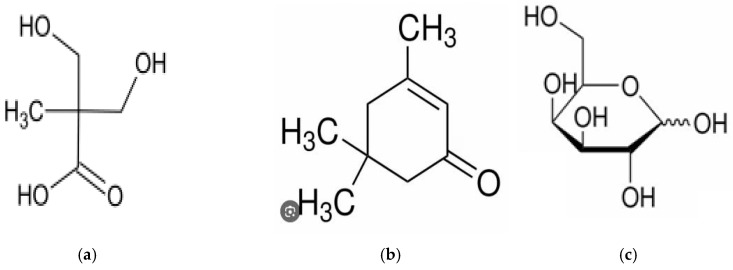

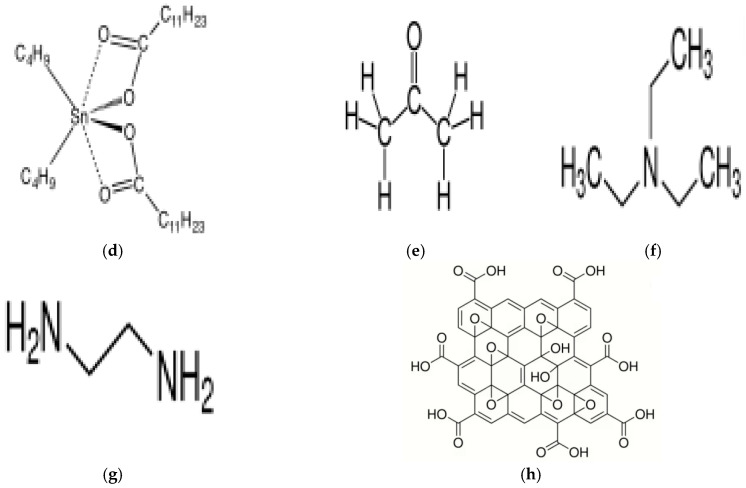
Molecular structure of standard functional group, (**a**) 2,2-Bis hydroxymethyl propionic acid, (**b**) Polypropylene glycol, (**c**) Isophorone, (**d**) Dibutyltin dilaurate, (**e**) Acetone, (**f**) Triethylamine, (**g**) Ethylenediamine [23], and (**h**) graphene Oxide nanocolloids.

All of the standard reagents shown in Figure 1a–h were prepared as follows. The reagents were from Sigma Aldrich. The molecule’s structure is shown below. Also, the synthesis reaction vessel was used with 100% SiO_2_ quartz crystals in laboratory conditions to remove any possible trace interference of ionic effects. All syntheses were performed at a temperature of 25 °C. Tap water was used for purification, repeatedly distilled with 18 M OHM resistance. The metal container was blocked to prevent the elution of impurities by using 100% stainless steel.

The reagents used were of 95.0% or higher purity, supplied by Aldrich Sigma Co. (St. Louis, MO, USA). Nevertheless, if necessary, further purification was also performed to remove any interfering effects. The molecular weight of (b)~(d) ranges from 58.6 to 1513 g/mol, while the density ranges between 0.72 and 1.05 g/cm^3^ at 20 °C. Controlling both the solubility and vapor pressure was possible for water dispersion and oil dispersion. In addition, it was also possible to synthesize these at both the melting point and boiling point of the isotherm. For these reasons, no other additional devices were used [23].

### 3.2. Synthetic Sequence

The synthesis order is as follows, according to reference [23]. The basic oil dispersion polymer mixing ratio was investigated through trials of a variety of methods. The statistically confirmed optimum molar ratio was examined by using reference methods. The temperature also maintained in the range of 80~85 °C to prevent high-temperature evaporation. The reaction time was activated within 8 h, and further optimal sequential substitutions were performed by using reference standard methods; the synthetic molar ratio, the optimal temperature, and other conditions were determined with reference to published research. However, the results optimized for physical functionality were increased via the addition of DMPA, which was then added at 80~85 °C for 6 h to allow for sufficient synthesis time. In addition, TRA and water were added to allow for stabilization at 40 °C in the same manner. TEA, EDA, and defamer were sequentially converted in order to enhance ductility, abrasion, resistance, and physicochemical bonding strength. The changed molar ratios were recorded, and the results of the bio physical and chemical properties of impact strength were subsequently combined.

### 3.3. Electrochemical Voltametric Analyzer

**Figure 2:**
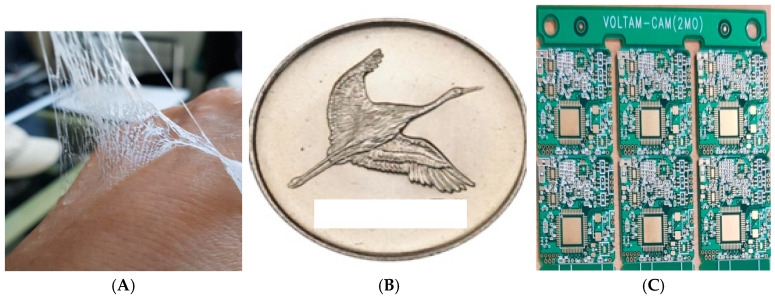
(**A**) A synthesized mimic skin tattoo film, (**B**) a 28 mm diameter coin like in C, and (**C**) a voltametric wearable WiFi workstation circuit with a coin size like that of B. Diagnostic COVID-19 antibody spike proteins are only responded to by antigen protein receptors.

Figure 2A shows a synthetic mimic skin tattoo film prototype coated on a back-hand sensing probe, which was 0.3 mm thick with working conductivity, and a counter and reference electrode, connected by a 0.3 mm copper wire within the terminal.

Figure 2A shows a real artificial skin film coated on the back of the experimenter’s hand. It is shrinkable and transparent. It is a conductive resin. It has strong ductility. Figure 2B shows a 28 mm coin sized-circuit. In this study, the voltametric diagnostic circuit was used as the bioelectrochemical analyzer-2. The workstations of our system are shown in Figure 2C and are used through WiFi. The wearable telemetric circuit, which was fabricated to a 3 cm × 28 mm coin size by telemetric control, has potential windows of 2.0 V~−2.0 V. For oxidation and reduction potential scanning, the detection current range is 1.01 × 0^−3^ A~1.01 × 0^−9^ A. Cyclic voltammetry, difference stripping voltammetry, and chronoamperometry control programs were used in 2D. The *x* axis is the potential windows and the *y* axis is the ionic strength. Furthermore, the diagnostic optimum conditions can be controlled, and the modified three-electrode system of the counter reference and antigen film electrode were used. Here, working probe fabrication was performed to achieve an antigen-immobilized DNA-linking synthetic mimic skin membrane.

### 3.4. COVID-19 Antigen-Immobilized Working Electrode

**Figure 3:**
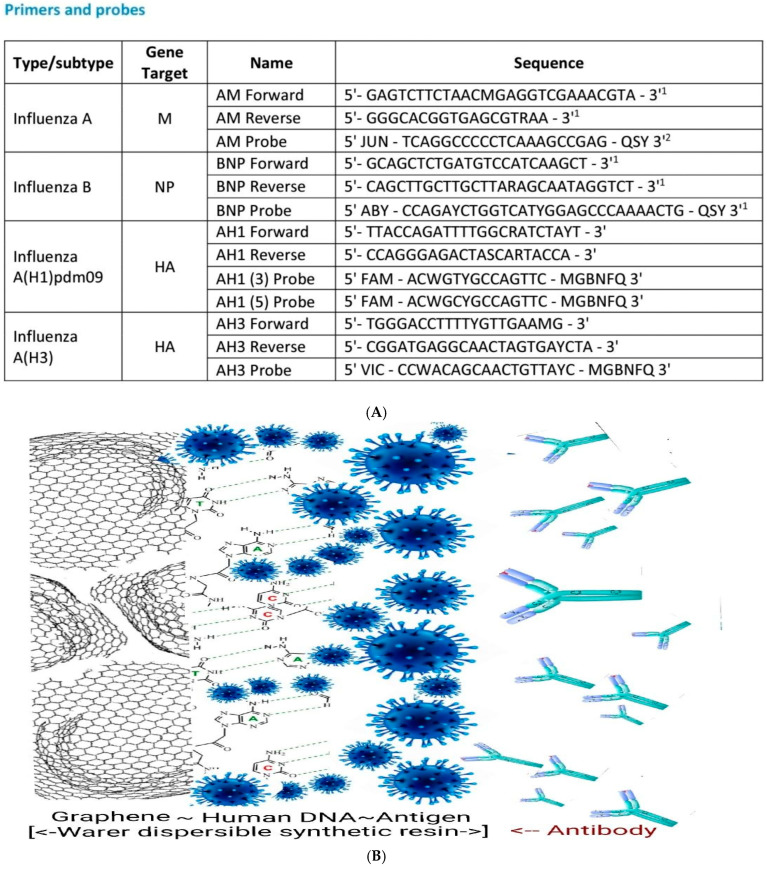

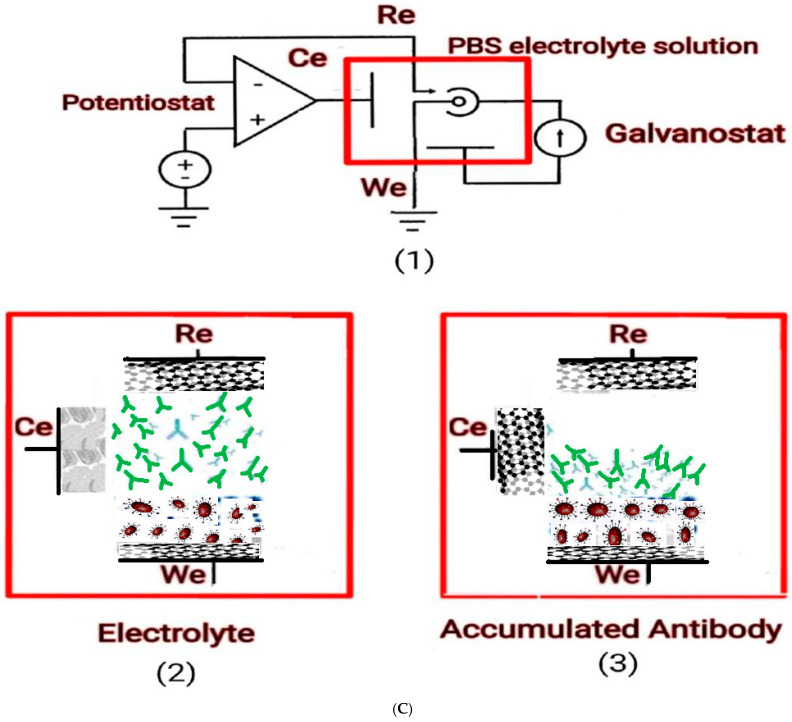
(**A**) Example DNA structure: antigen-immobilized COVID-19 combined probe for gene type sequence. (**B**) Schematic image of water-dispersible synthetic resin paste working electrode structure, with thickness of 0.5~1.0 µm (graphene, mixed platinum human DNA, COVID-19 antigen)<--redox titration layer-->patients’ blood antibody. (**C**) Voltametric amplification circuits of three-electrode cell (1); schematics of simulated redox titration before (2) and after (3).

A diagnostic working probe was fabricated by using a mixing ratio of graphene nano powder (2.0 g) and mineral oil (0.5 g). The water-dispersible synthetic resin (0.5 g) was mixed with 0.5 g antigen COVID-19 mixed plasma DNA, as shown in Figure 3A. The multi-layer is shown in Figure 3B of the working probe and in Figure 3C, it shows the redox electrode surface expansion reaction, the stripping accumulation is amplified by an exponential incress. The paste sensing surface was coated on the graphite crystal load with 0.5 mm diameter × 30 mm length and then connected to the voltametric working electrode terminal using a 0.35 mm pure copper enamel insulation coating wire. The counter and reference electrode were made using the same method with a graphite crystal load. The circuits are usable for wearable tattoo assays such as in vivo or in vitro and feeling sensing analysis.

Figure 3A shows an antigen mimic synthetic DNA sequence, where a 4-type mixed molecule was used as the working electrode immobilization paste. Figure 3B shows the working probe. It shows the overall schematic diagram of a multi-layer schematic image. The first layer is graphene powder, then the human DNA mixed platinum and COVID-19 antigen-immobilized activation layer, and the antibody redox titration current amplifier circuit is shown in Figure 3C. Figure 2C shows the circuit of the voltametric reaction cells, where (1) is the PBS electrolyte inserting skim, with a 5 mL solution buffer and (2) is the antibody 0.2 µL blood positive serum spike. Then, the cyclic voltametric accumulation time absorption skim is shown in (3), which includes the simulated antigen antibody molecular ionic redox titration skim. Under optimum conditions, the anodic and cathodic peak current obtained range within 1.0 × 10^−5^ A~1.0 × 10^−7^ A; more amplified signals can be obtained by using a stripping accumulation time with 1.0 × 10^−8^ A~1.0 × 10^−9^ A. The results can be used for in vivo or vitro detection.

## 4. Results and Discussion

### 4.1. Cyclic Voltametric Ionic Titration

The cyclic voltametric antigen antibody redox titration was performed by using an antigen-immobilized working electrode in purified water with 8 MΩ mega ohm resistance and PBS, Na_2_HPO_4_, and KH_2_PO_4_ buffer, using a 9% sodium chloride electrolyte at 5 mL at room temperature with graphite crystalline and a three-electrode system.

### 4.2. Cyclic Voltametric Redox Titration

**Figure 4:**
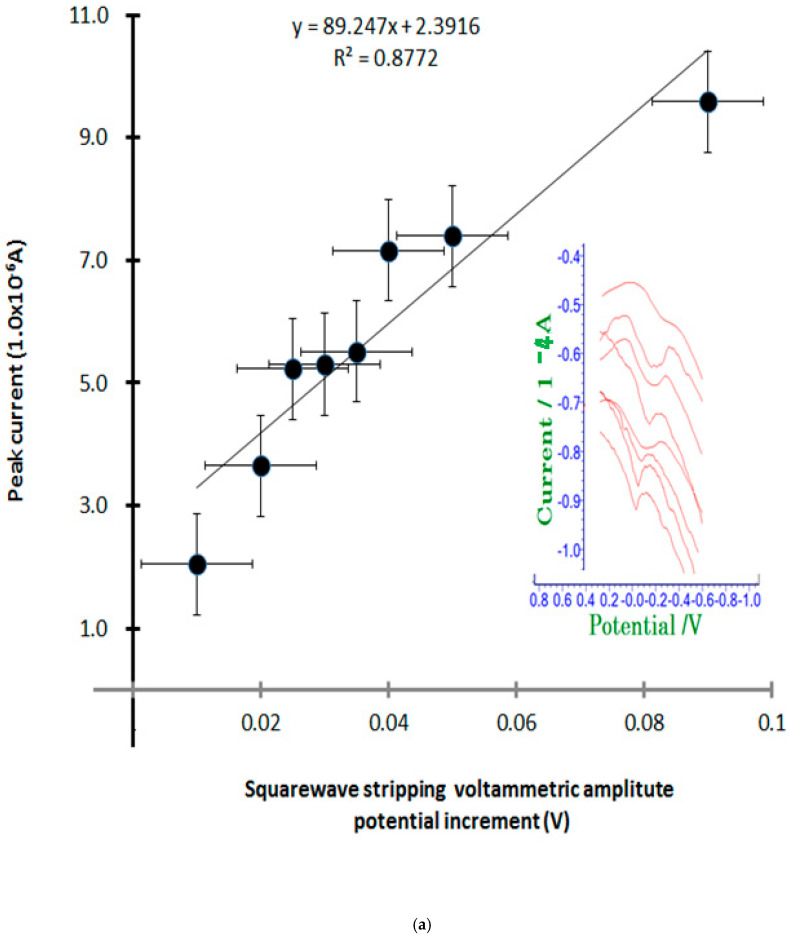

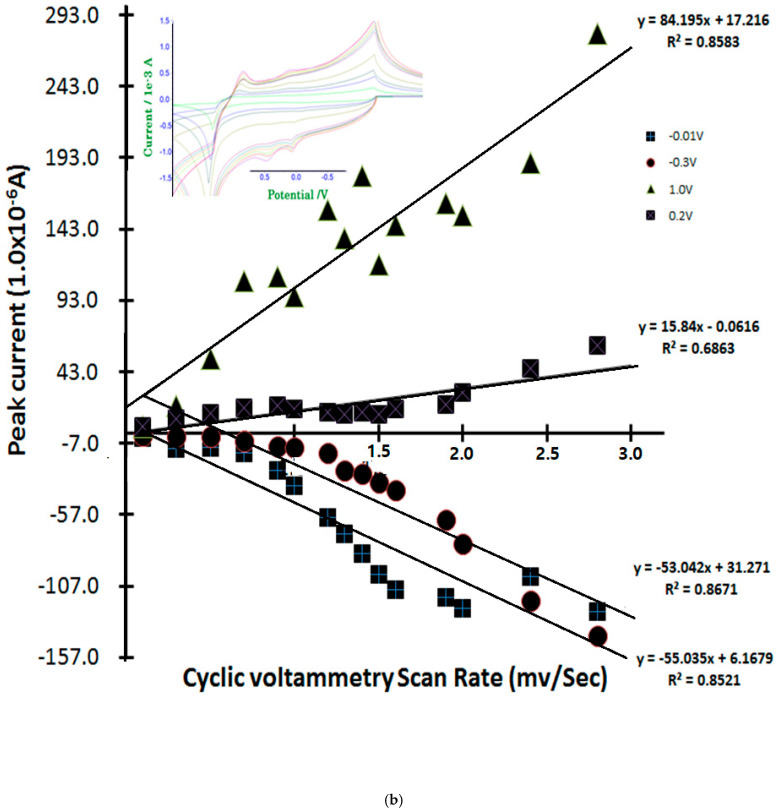

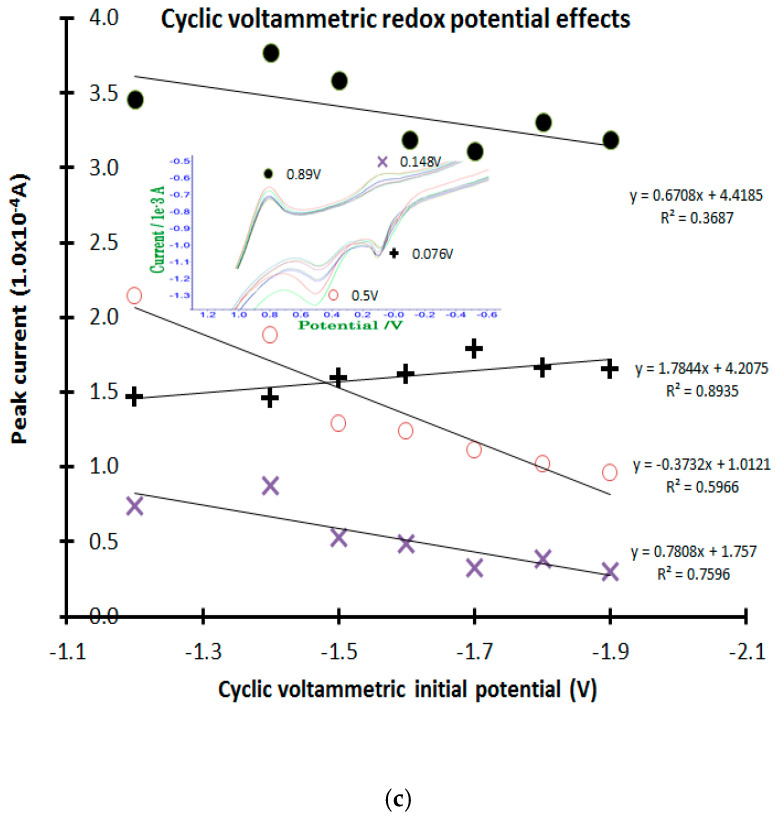
(**a**) Cyclic voltametric redox time variations over 13 steps within 20 s~140 s in PBS buffer with 3 mL electrolyte, 0.9 mL coronavirus antibody, and three-electrode system, using DNA immobilized working electrode. (**b**) Cyclic scan rate variations of (A) electrolyte. (**c**) Cyclic initial oxidation potential variations.

Figure 4a shows the analytical COVID-19 antibody cyclic accumulation time variations. Here, the results are related to the concentrated ionic activity, so a 13-step sequential peak current was examined. Then, it was combined with the inserted voltammograms, −2.0 V initial, 2.0 V feedback cyclic scanning, −0.2 V anodic, and 0.8 V cathodic peak current, obtained with 0.9 mL antibody-containing PBS electrolyte. During the 20~140 s accumulation time with redox scanning, the anodic peak is −0.1989 × 10^−6^ A~−16.15 × 10^−6^ A at −0.2 V potential windows, and R^2^ = 0.9005 is sensitive; also, the 0.8 V cathodic peak is 0.4848 × 10^−6^ A~−1.156 × 10^−6^ A, and a current appeared, for which the relative standard deviation R^2^ = 0.0129 is sensitive. Under these conditions, cyclic scan rate variations were examined. The results shown in Figure 4b for 0.1 mV/s~2.8 mV/s, with 25 step variations show that under optimum conditions, two peaks of anodic current for −0.01 V = −3.263 × 10^−6^ A~−124.7 × 10^−6^ A and −0.3 V = −1.362 × 10^−6^ A~−141.6 × 10^−6^ A appeared; here, the relative variation errors are shown to be inserted in the voltammograms. Also, cathodic peak obtained has two peaks. Figure 4c shows the cyclic scan rate variations of the initial potential within −1.2 V~−1.9 V; also, +0.076 V and +0.5 V oxidations appeared and +0.89 V + 0.148 V reduction peaks were obtained. Here, the linear equation for the +0.076 V oxidation peak is y = 0.7808X + 1.757, R^2^ = 0.7596; for the +0.5 V peak, it is y = −0.3737X + 1.01212, R^2^ = 0.5966; for the 0.89 V reduction peak, it is y = 1.7844X + 4.2075, R^2^ = 0.8935; and for 0.148 V, it is Y = 0.6708 + 4.4185, R^2^ = 0.3687. Then, stripping voltametric optimization was performed.

### 4.3. Stripping Voltametric Optimization

**Figure 5:**
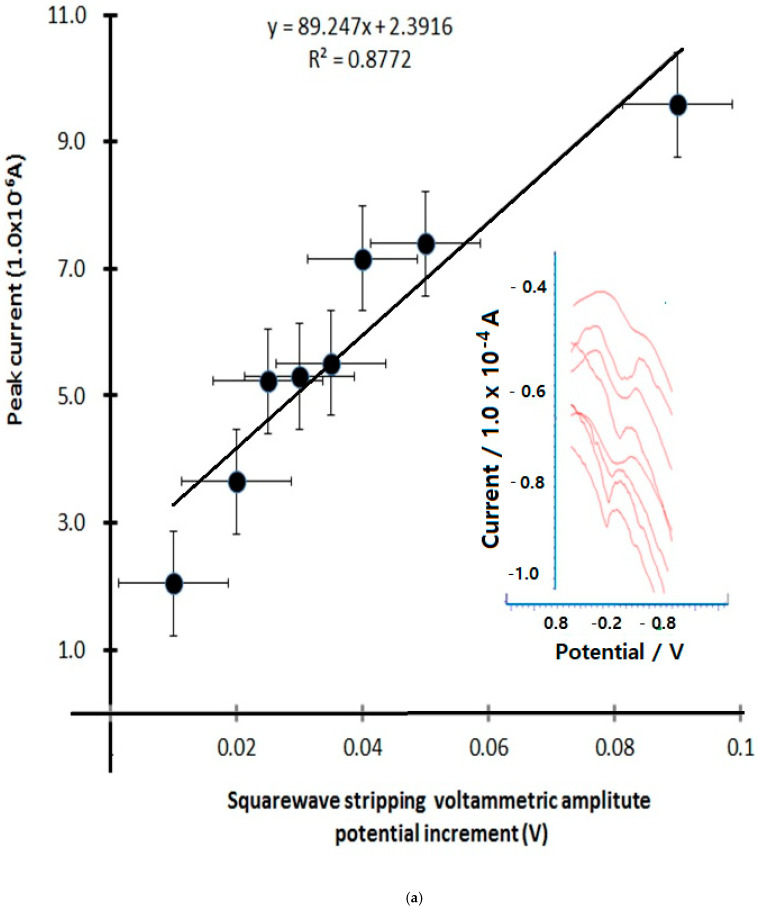

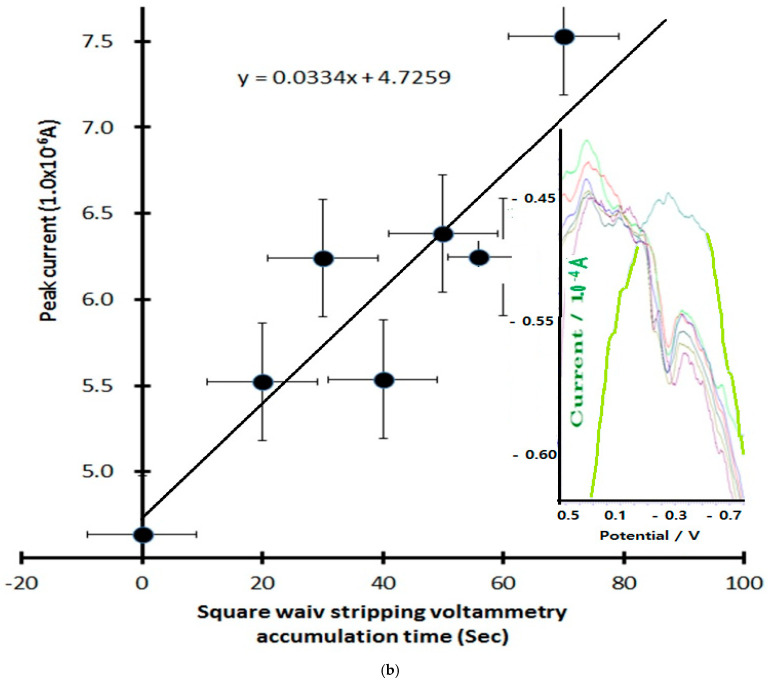
(**a**) Stripping voltametric anodic amplitude variation from 0.01 V to 0.09 V with eight intervals using 30 s accumulation time in 0.9 mL antigen spiked with PBS electrolyte constant; (**b**) stripping accumulation time variation from 0 s to 70 s with 7 intervals of anodic scanning, using (A) solution under same condition.

The stripping voltametric differential is dramatically increased by the sensitivity of ion titration. Therefore, only the following functions with the greatest influence were optimized, such as accumulation time change, current amplification rate, and redox titration. Figure 5a shows the results of the anodic stripping current amplitude variations with eight points, 0.01 mV, 0.02 mV, 0.025 mV, 0.03 mV, 0.035 mV, 0.04 mV, 0.05 mV, and 0.09 mV. In the real voltammograms shown in this figure, the sensitive linear current was increased from 2.024 × 10^−6^ A to 9.591 × 10^−6^ A, the linear equation is Y = 89.247X + 2.3916, and the relative standard deviations is R^2^ = 0.8772, so the optimum amplitude obtained is 0.06 mV. Under these conditions, anodic scanning time variations were examined at 0 s, 20 s, 30 s, 40 s, 50 s, 60 s, and 70 s, for which the voltammograms are shown in Figure 5b. The first peak is 4.637 × 10^−6^ A, which then linearly increases to 7.528 × 10^−6^ A; here, the working equation is dx/dy = 0.0334x, the intercept is 4.7259, and the anodic peak potential is fixed at −0.2 V. For these results, the optimum conditions obtained were at 30 s under these conditions. The final optimum conditions will affect the detection limits of the virus investigated, and thus the concentration spike was assessed according to mg~ug variation. The results show only the working curves and anodic scanning; analytical sensitivity comparisons for SARS-CoV-2 are shown in Table 2. The analytical limits of detection for seven SARS-CoV-2 assays using serial dilutions of pooled patient material quantified with droplet digital PCR are shown. Limits of detection ranged from ≤10 to 74 copies/mL for commercial high-throughput laboratory analyzers [24] and from 167 to 511 copies/mL for sample-to-answer [24] and point-of-care instruments (Abbott ID NOW). However, our developed method reached a detection limit of 10 µL, which is more sensitive than that of common methods [25].

**Figure 6:**
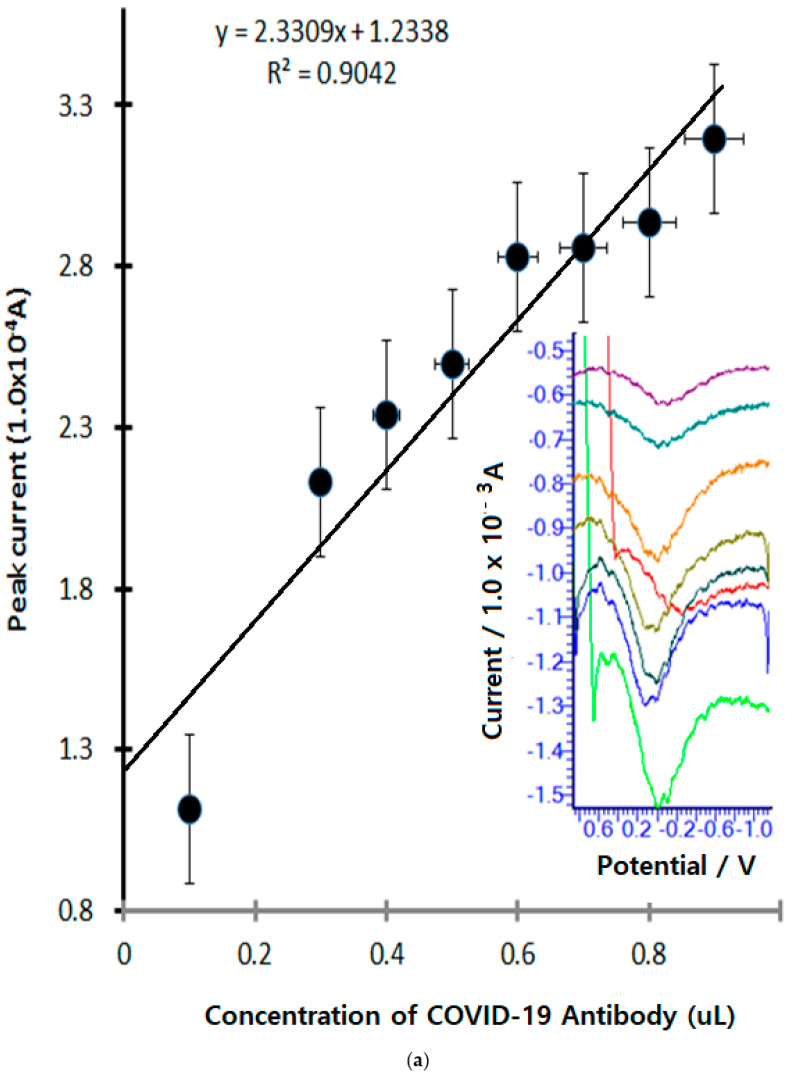

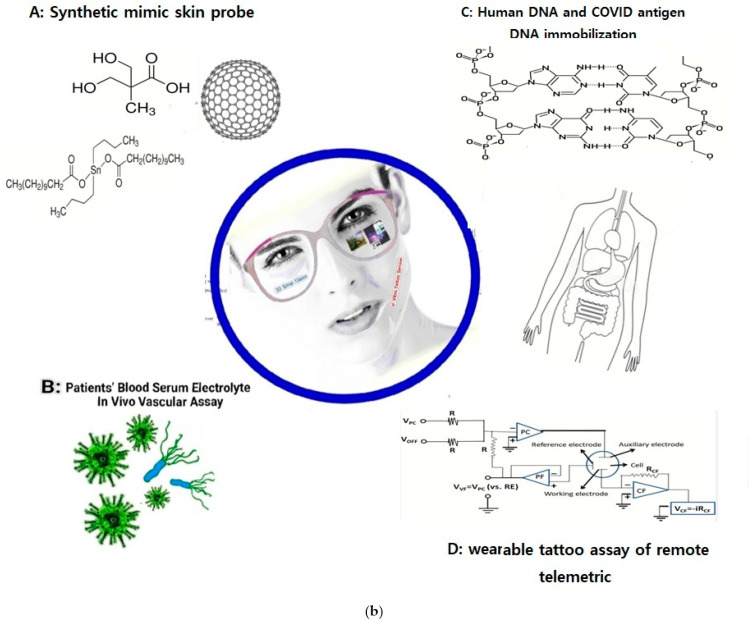
(**a**) Concentration effect of COVID-19 patients’ blood antibody serum spike for 0.1 µL, 0.2 µL, 0.3 µL, 0.4 µL, 0.5 µL, 0.6 µL, 0.7 µL, 0.8 µL, and 0.9 Ulin 2 mL PBS electrolyte using optimum parameters, with 30 s accumulation time, 0.06 mV amplitude, −2.0 V initial potential, and 2.0 V final potential used. (**b**) Oculus glass monitoring circuit in vitro and in vivo applications such as (A) synthetic mimic skin probe tattoo skin sensors, (B) patient blood serum electrolyte in vivo vascular assay, (C) human DNA and COVID-19 antigen DNA immobilization, (D) wearable tattoo assay for remote telemetric therapy, and glass monitoring control assays.

Using the final optimized stripping parameters, Figure 6a shows the effects of antibody concentration with 9-step spiking. The anodic scan with oxidized electric titration is shown in Figure 6; the first spike was added to 0.1 µL antibody in PBS electrolyte without any ion, with an obtained current of 1.113 × 10^−4^ A with 0.0 V potential windows. In the oxidized voltammogram, a small peak appears. With continued 0.3 µL~0.5 µL spiking, a 2.217 × 10^−4^ A~2.494 × 10^−4^ A peak current was obtained under this conditions. Figure 6b shows the oculus glass monitoring of in vitro and in vivo linking for applications such as synthetic artificial skin application. A paper was published on the synthesis of a water-dispersed human-friendly transparent skin sensor. It was synthesized at room temperature using graphene conductive carbon crystals, propylene hydrate, ethyl alcohol solvent, a soft reaction accelerator, and a room-temperature hardener. In vitro coating was used for skin tattooing and it was used as a three-electrode system. It was used as a voltage and current measurement sensor to amplify the signal of the antigen–antibody oxidation reaction. These sensors were suitable for disease diagnosis, virus diagnosis, heavy metal diagnosis, and biological signal amplification. It was possible to diagnose in vivo blood, urine, and body fluids within the human body. The micro sensor could be inserted into muscles, blood vessels, and organs without pain. Therefore, nerve signal detection, pain diagnosis, blood sugar assay, sound wave diagnosis, and joy, anger, and sorrow diagnosis are possible. Nerve current amplification can be linked to a remote unmanned control system. It can be linked with oculus glass monitoring. Therefore, multi-tasking and multi-user operations can be controlled. Figure 6b shows antigen–antibody diagnosis in the body. Antigen electrodes, antigen sensors, and signal amplification for in vivo virus diagnosis are possible. Therefore, virus amplification diagnosis at milli, micro, nano, and pico concentrations is possible. Additionally, an injection of cell-killing water is possible without organ damage. Linkage between voltammetry diagnostic equipment and oculus monitoring is possible. Therefore, wearable self-diagnosis sensors, self-treatment, unmanned diagnosis, drug injection, remote diagnosis treatment, etc., are possible. In addition, real-time human body information can be transmitted, enabling linkage with emergency treatment, micro-nano surgery, etc. The attached circuitry is a partial drawing designed by our laboratory. It was designed with two CPUs, an amplifier, memory, and WiFi transmission circuit. It is possible to convert more than 80% of ions in the periodic table at a cyclic redox voltage in the range of −2 v to 2 v. The ion potential can be amplified from milli to pico. Calculus-enabled 2D plane control is possible. Therefore, quantitative and qualitative methods can be performed. Nanopicomolar amplification of ion analysis is possible. The overall size of the circuit board ranges from the size of a coin to the DC power source. Therefore, we were able to design diagnostic equipment and sensors suitable for a wearable tattoo system.

## 5. Conclusions

Human DNA and an antigen DNA immobilized synthetic mimic skin probe responded to COVID-19 antibody ionic activity. Here, the redox titration current was amplified to 1.0 × 10^−3^ A~1.0 × 10^−4^ A. The diagnostic relative standard deviation attained was R^2^ = 0.9042, and the cyclic sensing potential was obtained at −0.01 V, −0.3 V for the anodic peak potential, and 1.0 V and 0.2 V for the cathodic peak potentials, which can be titrated for COVID-19 antibody detection in patient blood serum. However, the stripping time was only 0.0 V at the anodic peak and only appeared when 0.1 µL electrolyte was added. Under optimized conditions, analytical detection currents of the micro volume ranges were sought using a handmade mimic skin tattoo synthetic probe. The detection systems used were an electrochemical compact circuit like coin size portable telemetric analyzer. The analytical working time was only 30 s under optimum stripping conditions with 5 mL of electrolyte. The in vitro tattoo skin membrane probe can be used for virus recognition. Thus, a cyber networking human computer interfaced wearable tattoo assay is usable for remote telemetric therapy [25].

## Figures and Tables

**Table 1:** 2019 novel coronavirus approved real-time RT PCR detection primer sequence.

Names	Description	Oligonucleotide Sequence (5′Ç > 3′Ç)
2019-nCoV_N1-F	2019-nCoV_N1 forward primer	5′-Ç-GAC CCC AAA ATC AGC GAA AT-3′Ç
2019-nCoV_N1-R	2019-nCoV_N1 reverse primer	5′-TCT GGT TAC TGC CAG TTG AAT CTG-3′Ç
2019-nCoV_N1-P	2019-nCoV_N1 probe	5′-Ç-FAM-ACC CCG CAT TAC GTT TGG TGG ACC-BHQ1-3′Ç
2019-nCoV_N2-F	2019-nCoV_N2 forward primer	5′-Ç-TTA CAA ACA TTG GCC GCA AA-3′Ç
2019-nCoV_N2-R	2019-nCoV_N2 reverse primer	5′-Ç-GCG CGA CAT TCC GAA GAA-3′Ç
2019-nCoV_N2-P	2019-nCoV_N2 probe	5′-Ç-FAM-ACA ATT TGC CCC CAG CGC TTC AG-BHQ1-3′Ç
2019-nCoV_N3-F	2019-nCoV_N3 forward primer	5′-Ç-GGG AGC CTT GAA TAC ACC AAA A-3′Ç
2019-nCoV_N3-R	2019-nCoV_N3 reverse primer	5′-Ç-TGT AGC ACG ATT GCA GCA TTG-3′Ç
2019-nCoV_N3-P	2019-nCoV_N3 probe	5′-Ç-FAM-AYC ACA TTG GCA CCC GCA ATC CTG-BHQ1-3′Ç
RP-F	RNAse P forward primer	5′-Ç-AGA TTT GGA CCT GCG AGC G-3′Ç
RP-R	RNAse P reverse primer	5′-Ç-GAG CGG CTG TCT CCA CAA GT-3′Ç
RP-P	RNAse P	5′-Ç-FAM—TTC TGA CCT GAA GGC TCT GCG CG—BHQ-1-3′Ç

nCoV = novel coronavirus [22].

**Table 2:** Sample volumes tested for SARS-CoV-2 in new tab. ^a^ A 500 μL sample + 710 μL lysis buffer = 1210 μL input volume. ^b^ Sample-to-answer tests.

Assay	Minimum Input Vol (μL)	Sample Processed (μL)	Elution Vol (μL)	Eluate Added to Reaction (μL)	Effective Sample Vol (s) Tested (μL)
Abbott m2000	760	500	80	40	250
Roche Cobas	600	400	50	50	400
Panther Fusion	500 ^a^	360	50	5	25.4
DiaSorinSimplexa	— ^b^	—	—	—	50
GenMarkePlex	—	—	—	—	200
Abbott ID NOW	—	—	—	—	100 (target), 100 (internal)
CDC ABI 7500 (MP24)	350	200	100	5	10
CDC LC 480 (MP24)	350	200	100	5	10
CDC ABI 7500 (EZ1)	400	400	90	5	22.2
CDC LC 480 (EZ1)	400	400	90	5	22.2
Our Electrochemical method	10	10	0.5	0.05	10

## Data Availability

The original contributions presented in this study are included in the article. Further inquiries can be directed to the corresponding author.

## Abbreviations

COVID-19	Coronavirus disease 2019
WHO	World Health Organization
RT-PCR	Real-time polymerase chain reaction
nCoV	Novel coronavirus
PBS	Phosphate-buffered saline
